# Assessment of Preoperative Anxiety and Influencing Factors in Patients Undergoing Elective Surgery: An Observational Cross-Sectional Study

**DOI:** 10.3390/medicina60030403

**Published:** 2024-02-27

**Authors:** Juseok Oh, Wonjin Lee, Seunghee Ki, Jaewoo Suh, Seokwoo Hwang, Jeonghan Lee

**Affiliations:** 1 Department of Anesthesiology and Pain Medicine, Busan Paik Hospital, Inje University College of Medicine, Busan 47392, Republic of Korea; 093724@paik.ac.kr (J.O.); 2wonjin@hanmail.net (W.L.); emong0303@gmail.com (S.K.); jaewoo0918@naver.com (J.S.); tjrdn9852@gmail.com (S.H.); 2 Paik Institute for Clinical Research, Inje University College of Medicine, Busan 47392, Republic of Korea

**Keywords:** age, anxiety, education, general anesthesia, sex, prehabilitation

## Abstract

*Background and Objectives*: Preoperative anxiety is a common emotional response before elective surgery that influences postoperative outcomes and can increase analgesic requirements. However, clinicians frequently overlook these concerns. This study aimed to quantify preoperative anxiety and evaluate its association with patient-related factors. *Materials and Methods*: Anxiety levels were evaluated in adult patients awaiting elective surgery using the Korean-translated version of the Amsterdam Preoperative Anxiety and Information Scale (APAIS) and the State-Trait Anxiety Inventory-Korean YZ form (STAI-KYZ). The patients were also surveyed regarding the subjective causes of preoperative anxiety. *Results*: The study found that a total of 55 adult patients had a well-balanced subject distribution. Both questionnaires showed high internal consistency (Cronbach’s alpha values of 0.85 and 0.93). Significant correlations were observed in situational anxiety scores from the questionnaires, indicating differences between groups with high trait anxiety and those with normal anxiety levels (*p* < 0.05). Notably, female sex was the only patient-related factor that significantly affected the anxiety scores (*p* < 0.05). Furthermore, when considering additional patient factors stratified by sex, it became evident that younger females and females with prior general anesthesia experience displayed higher anxiety levels than their male counterparts. The most commonly reported subjective concern related to anesthesia was the fear of not regaining consciousness, followed by concerns about postoperative pain, intraoperative emergence, and other issues. *Conclusions*: This study confirms that being female is a significant risk factor for preoperative anxiety. Therefore, it is necessary to provide enhanced preoperative anxiolytic therapies, including preoperative patient education and other interventions, to individuals undergoing surgical procedures.

## 1. Introduction

Preoperative anxiety is a common and natural psychological response [1], with a prevalence ranging from 11 to 80%, according to the literature [2,3,4]. Interestingly, this anxiety is predominantly attributed to the prospect of anesthesia (65%) rather than the surgery itself (15%) [5]. Preoperative anxiety can be caused by several factors, including fear of postoperative pain, awakening during surgery, not waking up after surgery, postoperative nausea and vomiting (PONV), and loss of inhibition while under anesthesia [1,2,6,7]. As surgery is often considered one of the most unpleasant experiences a patient may encounter during their lifetime, preoperative anxiety can have a significant negative impact on their overall well-being.

Several studies have highlighted the importance of preoperative fitness in terms of physical, nutritional, and psychological aspects [8]. The preoperative period is a critical time for patients, as it can have both physical and psychological effects. Higher levels of anxiety can result in unstable intraoperative and postoperative hemodynamic parameters, such as arterial pressure, heart rate, and peripheral oxygen saturation [3,4]. These effects are particularly pronounced in the elderly [1]. Furthermore, perioperative anxiety can increase postoperative pain and the need for intraoperative anesthetics and postoperative analgesics [9,10]. One study found that higher trait anxiety was associated with an increased requirement for intravenous propofol during both anesthetic induction and maintenance [9]. This may be due to the confounding effect of anxiety on the autonomic nervous system [10]. Previous research has indicated that managing perioperative anxiety can reduce postoperative pain and analgesic requirements [11]. Furthermore, preoperative stress and anxiety may cause physiological changes that are associated with long-term postoperative morbidity and mortality [12]. Recently, there has been a growing emphasis on detailed perioperative management, including Enhanced Recovery After Surgery (ERAS) programs and preoperative conditioning (prehabilitation). The proactive preoperative program is designed to improve physical, nutritional, and psychological conditions including preoperative anxiety [8]. According to a systematic review, physical preoperative preparation improves postoperative morbidity and physical function. Additionally, there is also evidence suggesting that psychological conditioning affects surgical outcomes in both the short and long term [8,13].

Although negative consequences resulting from preoperative anxiety exist in perioperative management, healthcare providers often overlook them. Further research is needed to determine the contributing factors and solutions to preoperative anxiety to increase perioperative patient satisfaction. Understanding this psychophysical causal relationship can aid in perioperative management. A previous study suggested that sex is an independent predictor of higher preoperative anxiety [1,2,14,15,16,17]. However, the results for the remaining factors, such as age, previous history of anesthesia, and level of education, have been inconsistent across studies. For example, Lee et al. said that age was a predictor, Celik et al. said that education and type of anesthesia were predictors, and Badner et al. said that previous history was a predictor [15,16,17]. On the other hand, Bayrak et al. observed that there were no patient-related predictors for preoperative anxiety [4]. Based on these prior studies, we hypothesized that patient factors, including age, sex, previous history of anesthesia, and level of education, might impact levels of preoperative anxiety. In this study, we evaluated anxiety scores using the Korean-translated version of the Amsterdam Preoperative Anxiety and Information Scale (APAIS) and the State-Trait Anxiety Inventory-Korean YZ form (STAI-KYZ). Multiple studies [18,19] observed positive correlations between the APAIS and STAI questionnaires.

By using the anxiety questionnaires, the APAIS and STAI-KYZ, we also investigated the impact of patient characteristics, such as age, sex, academic background, and previous experiences with general anesthesia, on these scores. The main objective of this study was to analyze preoperative anxiety levels and identify factors contributing to patient demographics such as age, sex, academic background, and previous experience with general anesthesia. Additionally, the consistency of the Korean-translated versions of the questionnaires (the APAIS and STAI-KYZ) was evaluated. In addition, our secondary objectives involved investigating patients’ information desires and other potential contributing factors, as well as comprehending patients’ subjective concerns.

## 2. Materials and Methods

### 2.1. Study Design

This cross-sectional study was approved by the hospital’s Institutional Review Board (approval no. 17-0176, approved on 6 November 2017) and was conducted according to the ethical standards set by our institutional and national research committees. This study adhered to the principles of the 1964 Declaration of Helsinki and its subsequent amendments. Written informed consent was obtained from all patients before proceeding with interviews, including consent regarding the researcher, research purpose, and questionnaires, after which participants were asked to complete a questionnaire. The study participants were patients scheduled for surgery under general anesthesia. Included patients were adults over 19 years old (legal adults in the Republic of Korea), with American Society of Anesthesiologists (ASA) Physical Status Classification I to III (ASA I—a normal healthy patient, ASA II—a patient with mild systemic disease resulting in no functional limitations, and ASA III—a patient with severe systemic disease limiting but not incapacitating activity), and who could understand the study and questionnaires in the Korean language. The exclusion criteria were as follows: (1) unclear voluntary participation, (2) neuropsychological issues, (3) medical conditions or medication usage that could induce anxiety, (4) unclear communication abilities, and (5) incomplete survey responses.

Prior to the anxiety interview, each patient received explanations about anesthetic and surgical procedures. Patients heard identical explanations about general anesthesia. However, the type of surgery and its explanation differed between patients. The interviews were conducted at the patient’s bedside in the ward on the day before elective surgery. Participants were asked to complete a questionnaire. During the process, the patients were encouraged to ask questions to clarify their understanding. On average, each participant took approximately 10 min to complete the questionnaires. Patient demographics, including age, sex, previous experience with general anesthesia, and educational level, were collected directly from the patients and corroborated with the medical records of our medical center.

We used two self-reported structured questionnaires, the APAIS and STAI-KYZ. Demographic data (age, sex, history of general anesthesia, and educational background) were obtained using these self-completed questionnaires, and medical history was reviewed using electronic medical records from our hospital. Subjective responses related to anesthesia were collected. The survey tools were not altered according to the demographics.

### 2.2. Anxiety-Measuring Tools

The APAIS questionnaire, first introduced by Moerman et al. [20], is a widely used preoperative anxiety-measuring tool. Validation research has been conducted on the translation of the APAIS into various languages. This study used the Korean-translated version of the APAIS, which was meticulously translated to maintain semantic equivalence with the original version. The APAIS assesses preoperative anxiety related to anesthesia, the surgery itself, and the combined total of preoperative anxiety. It comprises six questions divided into anxiety related to anesthesia and surgical procedures (questions 1, 2, 4, and 5) and the need for information (information score) on anesthesia and surgical procedures (questions 3 and 6). The questions were graded using a five-point Likert scale, ranging from 1 = not at all to 5 = extremely [20]. The preoperative anxiety subscale scores ranged from 4 to 20 points, whereas the need-for-information subscale scores ranged from 2 to 10 points. A cutoff score of 11 points or more on the preoperative anxiety subscale showed a good predictive value with an acceptable balance between false-positive and false-negative patients [20]. Information scores were divided into three grades. Patients with scores between 2 and 4 on the information scale can be classified as having little or no information requirement, and those with scores between 8 and 10 have a higher information requirement [1,20]. In our study, the reliability coefficient (Cronbach’s ⍺) of the APAIS was 0.85. The preoperative anxiety subscales (questions 1, 2, 4, and 5) and need-for-information subscales (questions 3 and 6) had a Cronbach’s ⍺ of 0.86 and 0.76, respectively.

The STAI questionnaire is considered the gold standard tool for measuring state and trait anxieties owing to its well-established validity and reliability [10,21]. The STAI-KYZ is a translated version of the STAI-Y (Spielberger, 1983), which has been rebalanced by a number of positive and negative questions. The STAI questionnaire measures patients’ state and trait anxiety. State anxiety represents the current state of anxiety (situational). Trait anxiety refers to a relatively stable tendency or personality characteristic wherein an individual experiences a consistent level of anxiety across various situations and over time (baseline). The STAI-KYZ questionnaire consists of 40 questions divided into 20 questions each on state and trait anxiety. Each question was graded on a 4-point scale: 1 = not at all and 4 = extremely. Therefore, the total score ranged between 20 and 80. The questionnaires were divided into two groups of ten questions each. One group (questions 3, 4, 6, 7, 9, 12, 13, 14, 17, and 18) summed the scores of the questions, and the other group (questions 1, 2, 5, 8, 10, 11, 15, 16, 19, and 20) were negatively loaded and must be reversely scored. Kvaal et al. suggested a cutoff value of 55 for the STAI-KYZ to detect significant symptoms of anxiety in the elderly [1,22]. The state anxiety subscale of the STAI-KYZ had a Cronbach’s ⍺ of 0.93.

The questionnaires we used are shown in Supplementary Materials.

### 2.3. Statistical Analysis

The number of study subjects was based on the results of the study by Cornoiu et al. using the study’s mean and standard deviation (SD) (SD was 10.6 at stage 2, verbal group) to calculate the sample size [23]. The formula used to calculate the minimum sample size is as follows:

Minimum sample size = 2 ∗ (Z_(1/2α) + Z_β)^2 ∗ δ^2/(μ_T − μ_R)^2.

The required number of patients in each group was 28, with a significance level of 0.05 (Z_(1/2α) = 1.96), a power of 0.8 (Z_β = 0.842), and a hypothesized delta ((μ_T − μ_R)^2) of 8. The final study included 62 patients, allowing a dropout rate of 10%. They were divided into two groups, with an aim of 28 participants, each divided by their prior experience with general anesthesia.

Statistical analysis was performed using R (v4.3.2, R Core Team, 2023) and the Rstudio for Mac (v2023.09.1+494, Posit Software, 2023). Figures were created using the R extension ggplot2 (v3.4.2, H. Wickham et al., 2020). Dependent variables are expressed as the mean ± SD. Demographics and other categorical data are expressed as frequencies and percentages. ANOVA and *t*-tests were used to analyze the dependent variables depending on their categories. Correlation analysis (Pearson’s correlation coefficient) and simple linear regression, which involved questionnaire scores, were used to identify the relationship between each questionnaire. Statistical results are presented with 95% confidence intervals (CIs). Statistical significance was set at *p* < 0.05.

## 3. Results

**Figure 1:**
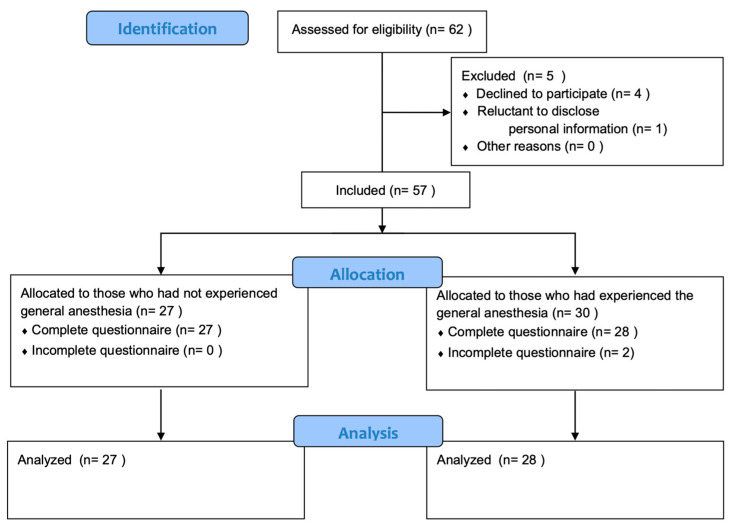
STROBE flow diagram for applying exclusion criteria and allocation: each group of 27 and 28 patients was allocated by prior general anesthesia experience. STROBE: Strengthening the Reporting of Observational Studies in Epidemiology.

Data were collected for 62 patients. After excluding five individuals who withdrew their consent for information disclosure and two who did not complete the survey, we identified seven dropouts. Consequently, we analyzed survey data from 27 participants in one group with no general anesthesia experience and 28 participants in the other group based on their experience with general anesthesia (Figure 1). We excluded the data on the remaining time before the surgery because for most of the patients we surveyed this was less than 1 week. The study subjects were evenly distributed in terms of sex and general anesthesia experience. Regarding the educational background of the study participants, the highest percentage (41.8%) was represented by individuals who were high school graduates. The detailed demographics of the study population are presented in Table 1.

**Figure 2:**
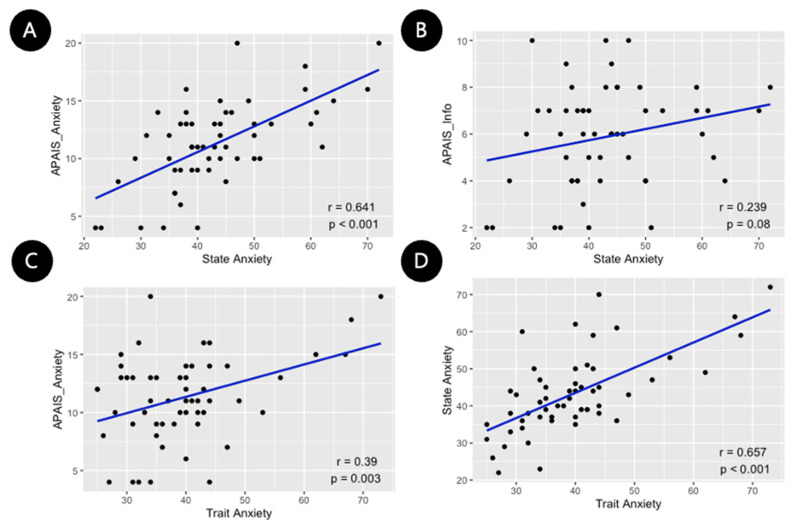
The scatter plot charts of the Pearson correlation coefficient. (**A**) The state anxiety and anxiety score of APAIS showed a significantly high positive correlation (r = 0.641, *p* < 0.001); (**B**) the state anxiety and information score of APAIS showed a low correlation (r = 0.239, *p* = 0.08); (**C**) the trait anxiety and anxiety score of APAIS showed a significant low correlation (r = 0.39, *p* = 0.003); and (**D**) the state and trait anxiety showed a significantly high positive correlation (r = 0.657, *p* < 0.001). APAIS: Amsterdam Preoperative Anxiety and Information Scale. APAIS_info: need-for-information subscore of APAIS (range from 2 to 10 points). APAIS_Anxiety: the degree of uneasiness about surgery and anesthesia in APAIS (range from 4 to 20 points). State anxiety: state anxiety score of the State-Trait Anxiety Inventory-Korean YZ form (STAI-KYZ). Trait anxiety: trait anxiety score of the STAI-KYZ.

The mean values of the APAIS and STAI-KYZ scores are shown in Table 2. A strong statistically significant correlation (r = 0.641, *p* < 0.001) was found between the state anxiety of the STAI-KYZ and the anxiety score of the APAIS (Figure 2A). Additionally, a moderate statistically significant correlation was found between the trait anxiety of the STAI-KYZ and the anxiety score of the APAIS (r = 0.39, *p* = 0.003) (Figure 2C). Furthermore, there was a significantly strong correlation between state and trait anxiety in the STAI-KYZ (r = 0.65, *p* < 0.001) (Figure 2D). The APAIS scores for anxiety and information were found to have a statistically significant moderate correlation (r = 0.4991, *p* < 0.001). However, there was no significant correlation found between the state and information scores of the APAIS (r = 0.239, *p* = 0.08) (Figure 2B) or between trait anxiety and information scores (r = 0.042 and *p* = 0.76).

**Figure 3:**
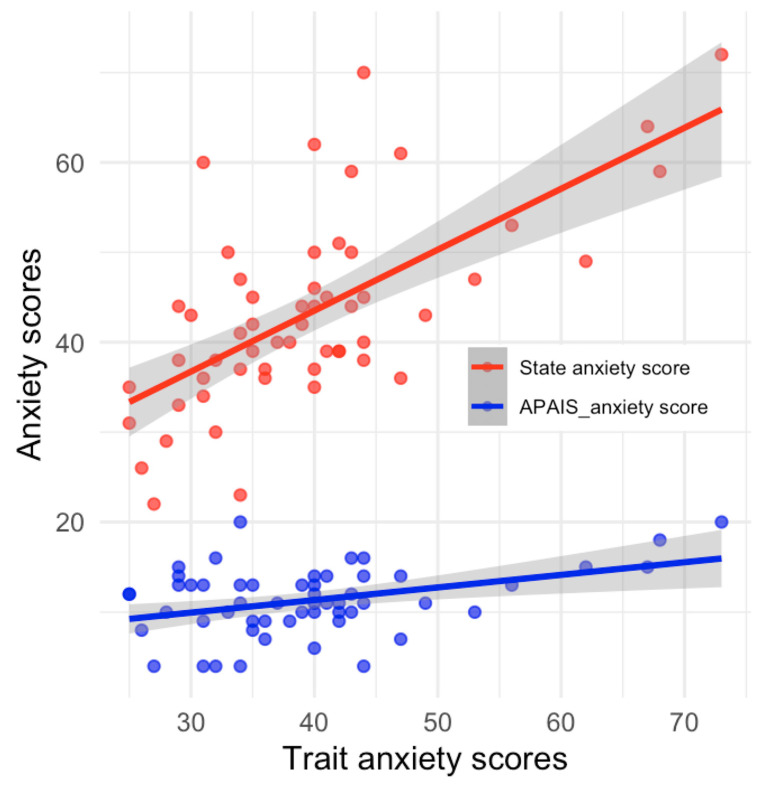
The association between trait anxiety score and state anxiety score of STAI-KYZ (red) and anxiety score of APAIS (blue). The ‘red’ solid line shows a simple linear regression of trait anxiety vs. state anxiety (state anxiety = 0.6781 ± trait anxiety + 16.3897, *p* < 0.001), and the gray shadow nearby represents a 95% confidence interval (CI). The ‘red’ dots show the distribution of the state anxiety score. The ‘blue’ solid line shows a simple linear regression of trait anxiety vs. anxiety score of APAIS (anxiety score of APAIS = 0.1396 ± trait anxiety + 5.7591, *p* = 0.003), and the gray shadow nearby represents 95% CI. The ‘blue’ dots show the distribution of the anxiety score of APAIS. APAIS: Amsterdam Preoperative Anxiety and Information Scale. APAIS_Anxiety score: the degree of uneasiness about surgery and anesthesia in APAIS (range from 4 to 20 points). State anxiety: state anxiety score of the State-Trait Anxiety Inventory-Korean YZ form (STAI-KYZ). Trait anxiety: trait anxiety score of the STAI-KYZ.

Kvaal et al. [22] established a cutoff for significant anxiety of 55. Patients were divided into two groups based on their trait anxiety level: a normal group with scores ranging from 20 to 54 points, and a significant anxiety group with a score of 55 points or higher. The number of patients in each group was 55 (91%) and 5 (9%), respectively. Table 3 displays the scores for state anxiety of the STAI-KYZ and the anxiety score and information score of the APAIS for each group divided by the trait anxiety score. The results presented in Figure 3 indicate that there is a positive correlation between trait anxiety and both state anxiety and anxiety scores on the APAIS. However, there was no statistically significant difference in the information score (5.8 ± 2.2 vs. 6.8 ± 1.6; *p* = 0.26).

The questionnaire scores were assessed based on age, sex, history of general anesthesia, and level of education. The anxiety scores of the APAIS and state anxiety scores of the STAI-KYZ were significantly higher in females than in males (9.8 ± 3.0 vs. 12.8 ± 3.9, *p* = 0.003 and 39.6 ± 8.8 vs. 46.8 ± 11.5, *p* = 0.011, respectively). Statistical analyses are presented in Table 4. There were no statistically significant differences in anxiety scores according to age, history of general anesthesia, or level of education. However, there were further divisions among females based on age, history of general anesthesia, and education level. Specifically, females who were younger or had prior anesthesia experience exhibited more anxiety than their male counterparts (Table 5). Furthermore, division among females based on education level did not yield a significant difference in anxiety scores of both the APAIS and STAI-KYZ. No statistically significant difference was observed in the groups when categorized by age, sex, history of general anesthesia, and level of education regarding the need for information about surgery and anesthesia.

In addition to administering the APAIS and STAI-KYZ questionnaires, we conducted a subjective inquiry to determine the primary preoperative concerns of our participants. Of the 55 participants surveyed, 31 shared subjective apprehensions. The majority of the participants (*n* = 17, 54.8%) expressed significant apprehensions about the possibility of not regaining consciousness after surgery. Other significant concerns reported were postoperative pain and complications (*n* = 6, 19.4%), emergence during surgery (*n* = 4, 12.9%), the operating room environment (*n* = 2, 6.4%), fear of anesthesia, and the surgical procedure itself (*n* = 1, 3.2%).

## 4. Discussion

The aim of our study was to determine the preoperative anxiety related to general anesthesia and surgical operation and to investigate patient factors such as age, sex, history of general anesthesia, and educational level. Our study design, consistent with previous research, provides additional support for reinforcing the existing evidence base and demonstrates consistent results in the Korean-translated versions of the questionnaires (the APAIS and STAI-KYZ).

The questionnaires showed high internal consistency, with Cronbach’s alpha values of 0.85 and 0.93, confirming the credibility of the Korean-translated versions of the questionnaires. The STAI-KYZ had been previously validated by Han et al. [24], and we observed correlations between the APAIS and the established STAI-KYZ. Similar to previous studies [18,19], our study found consistent results of statistically significant correlations between the state and trait anxiety scores of the STAI-KYZ and the anxiety scores of the APAIS. Our initial hypothesis was that some degree of anxiety might be positively correlated with the desire for information about anesthesia or surgical operations. The correlation analysis between the subscores of the APAIS (anxiety and information score) revealed a moderate correlation (r = 0.4991, *p* < 0.001), consistent with Moerman et al.’s study [20]. However, no relationship was found between the APAIS information score and the STAI-KYZ (state and trait anxiety) (*p* = 0.08 and *p* = 0.76, respectively). Although each survey has some degree of bias and it is impossible to determine superiority, our results suggest that both translated questionnaires are valid measures of situational anxiety related to anesthesia and surgery [25].

The state anxiety of the STAI-KYZ and APAIS (including both anxiety and information scores) was compared between the significant trait anxiety group (score above 55) and the normal group (others). The results showed statistically significant differences, with the significant trait anxiety group exhibiting higher state anxiety scores on the STAI-KYZ and anxiety scores on the APAIS (*p* < 0.01). Furthermore, we conducted a linear regression analysis of the state anxiety scores of the STAI-KYZ and anxiety score of the APAIS with the trait anxiety of the STAI-KYZ. The results were consistent with the previous study, confirming that a higher trait (baseline) anxiety level leads to higher state anxiety responses (situational) towards anesthesia and surgical operations. We also identified a tendency for the anxiety score of the APAIS to match this trend [26].

Several studies have concluded that the female sex is an elevated patient-related factor for perioperative anxiety [1,2,14,15,16,17]. Our research also concluded that the female sex was the sole patient-related factor. Other factors, including age, education level, and history of general anesthesia, did not affect preoperative anxiety scores. It is well known that women are more prone to anxiety disorders, except social anxiety disorders [27]. A systematic review showed that women are more likely to have an anxiety disorder in terms of psychosocial and biological factors and comorbidities [28]. Among the psychosocial factors analyzed, masculinity might be a protective factor against anxiety development, while femininity is the opposite [28]. Lim et al. hypothesized that men’s low tendency towards anxiety is due to their inability to easily reveal their feelings [1]. The sex-based groups were further subdivided by age, education level, and history of general anesthesia for further analysis (Table 5). Younger female patients displayed significantly higher levels of preoperative anxiety than their male counterparts (*p* < 0.05). Females, especially younger ones, have differences in anxiety thoughts compared to males. Bahrami et al. [29] found that younger females tend to perceive worrying as uncontrollable and adopt different strategies to cope with worries, leading to a higher prevalence of anxiety among this demographic group. In addition, our results were in accordance with those of a previous study [20] which found that females who experienced general anesthesia had more preoperative anxiety than their male counterparts (*p* < 0.05). The experience of negative events influences future sensitivity to anxiety. People typically react to anxiety by either diminishing it through familiarity or exacerbating it through negative feedback and anticipation of adverse experiences; this response varies among individuals. Supporting this, a previous study [30] suggested that individuals with higher trait anxiety tend to recall negative past events more anxiously. In preoperative settings, our research suggests that we should be more cautious, particularly with females who are relatively younger and have previously experienced general anesthesia. Notably, a significant disparity in anxiety related to upcoming events was observed among females, possibly stemming from a combination of psychological or biological factors [29].

It was proposed that higher levels of education may increase the desire for information, potentially leading to higher APAIS information scores. However, our study found no significant differences in APAIS information scores among those who had completed more advanced educational courses, those who had undergone a more advanced educational course, and those with other patient-related variables. This finding may be attributed to cultural factors in Korea. These include cultural modesty and nuances in the Korean language, which discourage asking questions, as well as cultural respect, politeness, and avoiding conflict with any professionals. Furthermore, this study found no patient factors that affected the information scores of the APAIS, in contrast to Moerman et al.’s original literature introducing the APAIS [20].

The preoperative concerns most frequently asked about were postoperative pain, emergence during surgery, and postoperative nausea and vomiting [2,6,7]. In contrast to the previously mentioned studies, our study found a pronounced preoperative fear of the inability to regain consciousness after surgery. This finding is consistent with a prior study conducted by Lim et al. [1]. Although there is a temporal difference between the two studies, they were both conducted at the same medical center, suggesting the possibility of cultural influences specific to Korea or the local community. This reflects a misunderstanding about anesthesia and surgery within Korean culture that undergoing surgery could lead to death. Although patients do not differ in their desire for information based on their personal characteristics, they often lack a clear understanding of anesthesia practices. Therefore, we acknowledge the need for enhanced anesthesia education during preoperative consultations, which has been somewhat neglected in the care of patients undergoing elective surgery.

In the field of anesthesiology, preoperative education and comprehensive preoperative anesthesia management are frequently undervalued. Preoperative education involves explaining the procedure, discussing the risks and benefits, providing perioperative care instructions, clarifying recovery expectations, and offering psychological support to ensure patients have a comprehensive understanding. Providing preoperative education sessions seems plausible for anxiolytic intervention. Several studies have demonstrated that preoperative education significantly reduces perioperative anxiety and postoperative pain [31,32,33]. Along with education, anxiolytic interventions such as pharmacological therapies (benzodiazepines, sympatholytics, anti-epileptics, and antidepressants) and other alternative therapies (aromatherapy, music therapy, and acupuncture) should also be considered [10].

This study has limitations as it did not take into account other patient-related factors, such as the details of the American Society of Anesthesiologists (ASA) Physical Status Classification, the type of surgery, and the complexity of surgery (major vs. minor). Caumo et al. reported a higher ASA Physical Status Classification and higher preoperative anxiety levels [14]. Our observations are limited by the admission of patients only one day before elective surgery. A previous study suggested that being admitted within a day before elective surgery is associated with preoperative anxiety [34]. Also, our study did not capture chronological changes in anxiety levels as the scheduled surgery date approached. To determine the efficacy of education and other preoperative anxiolytic therapies, future studies involving larger cohorts of patients and the assessment of standardized modalities will be essential.

## 5. Conclusions

In conclusion, our study highlighted patient-related factors that influence preoperative anxiety and demonstrated consistent results with the Korean translation of the questionnaires. It is noteworthy that a significant disparity in anxiety related to upcoming events was observed among females, which may be due to a combination of psychological and biological factors. Furthermore, preoperative anesthesia education is often overlooked in current anesthesiology practices. It is important to proactively implement preoperative preparation, including patient education, to reduce patient anxiety and improve perioperative patient outcomes. Therefore, anesthesiologists should exercise caution and provide anxiolytic interventions, especially for female patients and those with comorbid psychiatric disorders.

## Figures and Tables

**Table 1:** Demographic data of respondents (*n* = 55).

Demographics		Count (*n* = 55)	Percentage (%)
Age (yr)	19–30	13	23.6
	31–50	27	49.1
	51–65	12	21.8
	>65	3	5.5
Sex	Male	27	49.1
	Female	28	50.9
General anesthesia experience	Never	27	49.1
	More than once	28	50.9
Education level	Below high school	7	12.7
	High school graduate	23	41.8
	Some college	15	27.3
	Above undergraduate degree	10	18.2

**Table 2:** Descriptive statistics of anxiety and information scores before the surgery.

	Assessment Tool	Scores
APAIS	Sum	17.2 ± 5.2
	Anxiety score	11.3 ± 3.7
	Information score	5.9 ± 2.2
STAI-KYZ		
	State anxiety	43.3 ± 10.8
	Trait anxiety	39.6 ± 10.4

APAIS: Amsterdam Preoperative Anxiety and Information Scale. Sum: total sum of 6 APAIS questionnaires (range from 6 to 30 points). Anxiety score: the degree of uneasiness about surgery and anesthesia in APAIS (range from 4 to 20 points). Information score: need-for-information score in APAIS (range from 2 to 10 points). State anxiety: state anxiety (transitory emotional state) score of the State-Trait Anxiety Inventory-Korean YZ form (STAI-KYZ). Trait anxiety: trait anxiety score of the STAI-KYZ (a relatively stable tendency or personality characteristic is an individual’s consistent level of anxiety). Values are presented as mean ± SD.

**Table 3:** APAIS scores and state anxiety of STAI-KYZ compared with trait anxiety of STAI-KYZ divided by the cutoff value of 55.

Assessment Tool		STAI-KYZ—Trait Anxiety		
		20 to 54 (*n* = 50)	55 over (*n* = 5)	*p*-value
APAIS	Anxiety score	10.8 ± 3.5	16.2 ± 2.8	0.009 *
	Information score	5.8 ± 2.2	6.8 ± 1.6	0.26
STAI-KYZ	State anxiety	41.6 ± 9.6	59 ± 9.0	0.009 *

The two groups divided by trait anxiety at the cutoff value of 55 were compared by the *t*-test. APAIS: Amsterdam Preoperative Anxiety and Information Scale. Sum: the total sum of 6 APAIS questionnaires (range from 6 to 30 points). Anxiety score: the degree of uneasiness about surgery and anesthesia in APAIS (range from 4 to 20 points). Information score: need-for-information score in APAIS (range from 2 to 10 points). State anxiety: state anxiety (transitory emotional state) score of the State-Trait Anxiety Inventory-Korean YZ form (STAI-KYZ). Trait anxiety: trait anxiety score of the STAI-KYZ (a relatively stable tendency or personality characteristic is an individual’s consistent level of anxiety). Values are presented as mean ± SD. * *p* < 0.05.

**Table 4:** Scores of anxiety questionnaires compared by sex.

Assessment Tool		Sex		
		Male (*n* = 27)	Female (*n* = 28)	*p*-Value
APAIS	Anxiety score	9.8 ± 3.0	12.8 ± 3.9	0.003 *
	Information score	5.8 ± 2.1	6.0 ± 2.2	0.80
STAI-KYZ	State anxiety	39.6 ± 8.8	46.8 ± 11.5	0.011 *
	Trait anxiety	37.6 ± 8.4	41.6 ± 11.5	0.16

The two groups divided by sex were compared by *t*-test. APAIS: Amsterdam Preoperative Anxiety and Information Scale. Sum: the total sum of 6 APAIS questionnaires (range from 6 to 30 points). Anxiety score: the degree of uneasiness about surgery and anesthesia in APAIS (range from 4 to 20 points). Information score: need-for-information score in APAIS (range from 2 to 10 points). State anxiety: state anxiety (transitory emotional state) score of the State-Trait Anxiety Inventory-Korean YZ form (STAI-KYZ). Trait anxiety: trait anxiety score of the STAI-KYZ (a relatively stable tendency or personality characteristic is an individual’s consistent level of anxiety). Values are presented as mean ± SD. * *p* < 0.05.

**Table 5:** Comparison of anxiety scores of groups divided by sex and further categorized by age and history of general anesthesia.

Anxiety Score of APAIS		Sex		
		Male (*n* = 27)	Female (*n* = 28)	*p*-Value
History of anesthesia	Never (*n* = 27)	10.4 ± 3.2 (*n* = 16)	13.1 ± 3.9 (*n* = 11)	0.06
	More than once (*n* = 28)	8.91 ± 2.4 (*n* = 11)	12.5 ± 3.9 (*n* = 17)	0.011 *
State anxiety of STAI-KYZ				
				*p*-value
History of anesthesia	Never (*n* = 27)	41 ± 10.4 (*n* = 16)	46.8 ± 11.7 (*n* = 11)	0.19
	More than once (*n* = 28)	37.5 ± 5.6 (*n* = 11)	46.8 ± 11.7 (*n* = 17)	0.022 *
Anxiety score of APAIS				
				*p*-value
Age	19–30 (*n* = 13)	9.5 ± 3.5 (*n* = 10)	15 ± 1.7 (*n* = 3)	0.026 *
	31–50 (*n* = 27)	9.22 ± 2.9 (*n* = 9)	12.5 ± 3.6 (*n* = 18)	0.027 *
	51–65 (*n* = 12)	10.7 ± 2.8 (*n* = 7)	11.6 ± 6.0 (*n* = 5)	0.73
	65- (*n* = 3)	11 (*n* = 1)	14.5 + 0.7 (*n* = 2)	0.15
State anxiety of STAI-KYZ				
				*p*-value
Age	19–30 (*n* = 13)	37.9 ± 6.3 (*n* = 10)	56 ± 15.7 (*n* = 3)	0.010 *
	31–50 (*n* = 27)	38.7 ± 10.4 (*n* = 9)	46.7 ± 9.9 (*n* = 18)	0.10
	51–65 (*n* = 12)	41.4 ± 10.9 (*n* = 7)	40 ± 11.7 (*n* = 5)	0.83
	65- (*n* = 3)	43 (*n* = 1)	51 ± 18.4 (*n* = 2)	0.78

The groups were categorized by sex and compared by *t*-test. APAIS: Amsterdam Preoperative Anxiety and Information Scale. Anxiety score of APAIS: the degree of uneasiness about surgery and anesthesia in APAIS (range from 4 to 20 points). State anxiety of STAI-KYZ: state anxiety (transitory emotional state) score of the State-Trait Anxiety Inventory-Korean YZ form. Values are presented as mean ± SD. The unit of age is years. * *p* < 0.05.

## Data Availability

The research data are available on request.

## Supplementary Materials

The following supporting information can be downloaded at: https://www.mdpi.com/article/10.3390/medicina60030403/s1.

## References

[1] Lim S., Oh Y., Cho K., Kim M., Moon S., Ki S. (2020). The Question of Preoperative Anxiety and Depression in Older Patients and Family Protectors. Anesth. Pain Med..

[2] Mavridou P., Dimitriou V., Manataki A., Arnaoutoglou E., Papadopoulos G. (2013). Patient’s Anxiety and Fear of Anesthesia: Effect of Gender, Age, Education, and Previous Experience of Anesthesia. A Survey of 400 Patients. J. Anesth..

[3] Tadesse M., Ahmed S., Regassa T., Girma T., Mohammed A. (2022). The Hemodynamic Impacts of Preoperative Anxiety among Patients Undergoing Elective Surgery: An Institution-Based Prospective Cohort Study. Int. J. Surg. Open.

[4] Bayrak A., Sagiroglu G., Copuroglu E. (2019). Effects of Preoperative Anxiety on Intraoperative Hemodynamics and Postoperative Pain. J. Coll. Physicians Surg. Pak..

[5] Ramsay M.A.E., Macaluso A., Hein T.H.A., Cancemi E. (1998). Use of Remifentanil in Patients Breathing Spontaneously during Monitored Anesthesia Care and in the Management of Acute Postoperative Care. Anesthesiology.

[6] McCleane G.J., Cooper R. (1990). The Nature of Pre-Operative Anxiety. Anaesthesia.

[7] Ruhaiyem M., Alshehri A., Saade M., Shoabi T., Zahoor H., Tawfeeq N. (2016). Fear of Going under General Anesthesia: A Cross-Sectional Study. Saudi J. Anaesth..

[8] Le Roy B., Selvy M., Slim K. (2016). The Concept of Prehabilitation: What the Surgeon Needs to Know?. J. Visc. Surg..

[9] Maranets I., Kain Z.N. (1999). Preoperative Anxiety and Intraoperative Anesthetic Requirements. Anesth. Analg..

[10] Baagil H., Baagil H., Gerbershagen M.U. (2023). Preoperative Anxiety Impact on Anesthetic and Analgesic Use. Medicina.

[11] Dunn L.K., Durieux M.E., Fernández L.G., Tsang S., Smith-Straesser E.E., Jhaveri H.F., Spanos S.P., Thames M.R., Spencer C.D., Lloyd A. (2018). Influence of Catastrophizing, Anxiety, and Depression on in-Hospital Opioid Consumption, Pain, and Quality of Recovery after Adult Spine Surgery. J. Neurosurg. Spine.

[12] Basak F., Hasbahceci M., Guner S., Sisik A., Acar A., Yucel M., Kilic A., Bas G. (2015). Prediction of Anxiety and Depression in General Surgery Inpatients: A Prospective Cohort Study of 200 Consecutive Patients. Int. J. Surg..

[13] Levett D.Z.H., Grimmett C. (2019). Psychological Factors, Prehabilitation and Surgical Outcomes: Evidence and Future Directions. Anaesthesia.

[14] Caumo W., Schmidt A.P., Schmidt A., Schneider C.N., Schneider C.N., Schneider C.N., Bergmann J., Iwamoto C.W., Bandeira D.R., Ferreira M.B.C. (2001). Risk Factors for Preoperative Anxiety in Adults. Acta Anaesthesiol. Scand..

[15] Celik F., Edipoglu I.S. (2018). Evaluation of Preoperative Anxiety and Fear of Anesthesia Using APAIS Score. Eur. J. Med. Res..

[16] Lee J.-S., Park Y.-M., Ha K.-Y., Cho S.-W., Bak G.-H., Kim K.-W. (2016). Preoperative Anxiety about Spinal Surgery under General Anesthesia. Eur. Spine J..

[17] Badner N.H., Nielson W.R., Munk S., Kwiatkowska C., Gelb A.W. (1990). Preoperative Anxiety: Detection and Contributing Factors. Can. J. Anaesth..

[18] Boker A., Brownell L., Donen N. (2002). The Amsterdam Preoperative Anxiety and Information Scale Provides a Simple and Reliable Measure of Preoperative Anxiety. Can. J. Anaesth..

[19] Turzáková J. (2014). Amsterdam Preoperative Anxiety and Information Scale Validation Study. Anxiety and Coping in Hospitalized Patients. Measurement Tools and Findings.

[20] Moerman N., van Dam F.S.A.M., Muller M.J., Muller M.J., Muller M.J., Oosting H. (1996). The Amsterdam Preoperative Anxiety and Information Scale (APAIS). Anesth. Analg..

[21] Spielberger C.D., Gorsuch R.L., Lushene R.E. (1970). STAI Manual for the State-Trait Anxiety Inventory (“Self-Evaluation Questionnaire”).

[22] Kvaal K., Ulstein I., Nordhus I.H., Engedal K. (2005). The Spielberger State-Trait Anxiety Inventory (STAI): The State Scale in Detecting Mental Disorders in Geriatric Patients. Int. J. Geriat. Psychiatry.

[23] Cornoiu A., Beischer A.D., Donnan L., Graves S., de Steiger R. (2011). Multimedia Patient Education to Assist the Informed Consent Process for Knee Arthroscopy. ANZ J. Surg..

[24] Hahn D.-W., Lee C.H., Chon K.K. (1996). Korean Adaptation of Spielberger’s STAI (K-STAI). Korean J. Psychol. Health.

[25] Kim Y.S., Shin W.J., Shin J.C., Shim J.H., Jeon W.J., Cho S.Y., Yeom J.H., Kim K.H. (2007). Does the Desire to Know about Information Related to Anesthesia and Surgery Differ according to the Coping Style Classified by the Amsterdam Preoperative Anxiety and Information Scale?. Korean J. Anesthesiol..

[26] Meijer J. (2001). Stress in the Relation between Trait and State Anxiety. Psychol. Rep..

[27] McLean C.P., Asnaani A., Litz B.T., Hofmann S.G. (2011). Gender Differences in Anxiety Disorders: Prevalence, Course of Illness, Comorbidity and Burden of Illness. J. Psychiatr. Res..

[28] Farhane-Medina N.Z., Luque B., Tabernero C., Castillo-Mayén R. (2022). Factors Associated with Gender and Sex Differences in Anxiety Prevalence and Comorbidity: A Systematic Review. Sci. Prog..

[29] Bahrami F., Yousefi N. (2011). Females Are More Anxious Than Males: A Metacognitive Perspective. Iran. J. Psychiatry Behav. Sci..

[30] Toffalini E., Mirandola C., Coli T., Cornoldi C. (2015). High Trait Anxiety Increases Inferential False Memories for Negative (but Not Positive) Emotional Events. Personal. Individ. Differ..

[31] Feninets V., Adamakidou T., Mantzorou M., Mastrogiannis D., Govina O., Tsiou C. (2022). The Effect of Preoperative Educational Intervention on Anxiety and Pain of Patients Undergoing Spinal Decompression Surgery: A Pilot Randomized Controlled Study. Cureus.

[32] Asilioglu K., Celik S.S. (2004). The Effect of Preoperative Education on Anxiety of Open Cardiac Surgery Patients. Patient Educ. Couns..

[33] Ahmed A.K., Mahmood N.A. (2023). The Impact of Preoperative Education on Reducing Anxiety among Patients Undergoing Elective Surgery at Shahidan Qaladze Teaching Hospital in Kurdistan Region/Iraq. Mosul J. Nurs..

[34] Shawahna R., Jaber M., Maqboul I., Hijaz H., Tebi M., Ahmed N.A.-S., Shabello Z. (2023). Prevalence of Preoperative Anxiety among Hospitalized Patients in a Developing Country: A Study of Associated Factors. Perioper. Med..

