# Supplementary material for: Assessment of Preoperative Anxiety and Influencing Factors in Patients Undergoing Elective Surgery: An Observational Cross-Sectional Study
Source: Medicina (Kaunas). 2024 Feb 27;60(3):403. doi: 10.3390/medicina60030403 (PMC10972132; doi:10.3390/medicina60030403)
Supplement: Supplementary file 1 [file medicina-60-00403-s001.zip › medicina-2888048-supplementary.pdf]

Questionnaire form

Attention: This survey is only being conducted voluntarily. Please check the appropriate box below.

1. What is your age?
   ( ) 19 – 30 years old
   ( ) 31 – 50 years old
   ( ) 51 – 65 years old
   ( ) 66 years and over
   ( ) I don't want to answer this question.

2. What is your gender?
   ( ) Male
   ( ) Female
   ( ) I don't want to answer this question.

3. When will you be having surgery or anesthesia?
   ( ) Tomorrow
   ( ) Within a week
   ( ) Within a month
   ( ) More than a month from now
   ( ) I don't want to answer this question.

4. What is your educational level?
   ( ) Below high school
   ( ) High school graduate
   ( ) Some college
   ( ) above undergraduate degree
   ( ) I don't want to answer this question.

5. Have you had general anesthesia in the past?
   ( ) Never, 0
   ( ) More than once
   ( ) I don't want to answer this question.

6. What makes you most anxious about going under general anesthesia?

   ____

the Amsterdam Preoperative and Information Scale (APAIS)

Questions	The Amsterdam preoperative Anxiety and information scale				
	1	2	3	4	5
1. I am worried about the anesthetic					
2. The anesthetic is on my mind continually					
3. I would like to know as much as possible about the anesthetic					
4. I am worried about the procedure					
5. The procedure is on my mind continually					
6. I would like to know as much as possible about the procedure					
1: Not at all, 2: Somewhat, 3: Moderate, 4: Moderately high, 5: Extremely					

the Amsterdam Preoperative and Information Scale (APAIS)

Self-evaluation Questionnaire I

Instruction: Below are statements that people use to describe themselves. Read each statement and circle the number to the right based on the **degree to which it best describes how you are feeling at this very moment.** There are no right or wrong answers to these questions. Don't spend too much time answering any one question, just circle the number to the right based on the degree to which you think it best describes how you're feeling right now.

	Not at all	Somewhat	Moderaterly so	Very much so
1. I feel calm____				
2. I feel secure____				
3. I am tense____				
4. I feel strained____				
5. I feel at ease____				
6. I feel upset____				
7. I am presently worrying over possible misfortunes_____				
8. I feel satisfied____				
9. I feel frightened____				
10. I feel comfortable____				
11. I feel self-confident____				
12. I feel nervous____				
13. I am jittery____				
14. I feel indecisive____				
15. I am relaxed___				
16. I feel content____				
17. I am worried____				
18. I feel confused____				
19. I feel steady____				
20. I feel pleasant _____				

1 = not at all; 2 = somewhat; 3 = moderately so; 4 = very much so

Self-evaluation questionnaire 2

Instruction: Below are statements that people use to describe themselves. Read each statement and circle the number to the right based **on the degree to which you think it best describes your personality tendencies that you often feel on a daily basis**. There are no right or wrong answers to these questions. Don't spend too much time answering any one question, just circle the number to the right based on the degree to which you think it best describes how you're feeling right now.

	Not at all	Somewhat	Moderately so	Very much so
21. I feel pleasant____				
22. I feel nervous and restless____				
23. I feel satisfied with myself____				
24. I wish I could be as happy as others seem to be____				
25. I feel like a failure____				
26. I feel rested____				
27. I am "calm, cool, and collected____ "				
28. I feel that difficulties are piling up so that I cannot overcome them____				
29. I worry too much over something that really doesn't matter____				
30. I am happy____				
31. I have disturbing thoughts____				
32. I lack self-confidence____				
33. I feel secure____				
34. I make decisions easily____				
35. I feel inadequate_____				
36. I am content____				
37. Some unimportant thought runs through my mind and bothers me____				
38. I take disappointments so keenly that I can't put them out of my mind____				
39. I am a steady person____				
40. I get in a state of tension or turmoil as I think over my recent concerns and interests _____				

1 = not at all; 2 = somewhat; 3 = moderately so; 4 = very much so
